# Asuc_0142 of *Actinobacillus succinogenes* 130Z is the l-aspartate/C4-dicarboxylate exchanger DcuA

**DOI:** 10.1099/mic.0.001411

**Published:** 2023-10-31

**Authors:** Young Bin Cho, Ji Won Park, Gottfried Unden, Ok Bin Kim

**Affiliations:** ^1^​ Division of EcoScience and Interdisciplinary Program of EcoCreative, Graduate School, Ewha Womans University, Seoul, 03760, Republic of Korea; ^2^​ Institute for Molecular Physiology (IMP), Microbiology and Biotechnology, Johannes Gutenberg-University, Biozentrum II, Hanns-Dieter-Hüsch-Weg 17, 55128 Mainz, Germany; ^3^​ Department of Life Science, Ewha Womans University, Seoul, 03760, Republic of Korea

**Keywords:** *Actinobacillus succinogenes*, C4DC, DcuA, L-aspartate, bovine rumen

## Abstract

Anaerobic bacteria often use antiporters DcuB (malate/succinate antiport) or DcuA (l-aspartate/succinate antiport) for the excretion of succinate during fumarate respiration. The rumen bacterium *Actinobacillus succinogenes* is able to produce large amounts of succinate by fumarate respiration, using the DcuB-type transporter DcuE for l-malate/succinate antiport. Asuc_0142 was annotated as a second DcuB-type transporter. Deletion of Asuc_0142 decreased the uptake rate for l-[^14^C]aspartate into *A. succinogenes* cells. Properties of transport by heterologously expressed Asuc_0142 were investigated in an *Escherichia coli* mutant deficient of anaerobic C4DC transporters. Expression of Asuc_0142 resulted in high uptake activity for l-[^14^C]fumarate or l-[^14^C]aspartate, but the former showed a strong competitive inhibition by l-aspartate. In *E. coli* loaded with l-[^14^C]aspartate, [^14^C]succinate or [^14^C]fumarate, extracellular C4DCs initiated excretion of the intracellular substrates, with a preference for l-aspartate_ex_/succinate_in_ or l-aspartate_ex_/fumarate_in_ antiport. These findings indicate that Asuc_0142 represents a DcuA-type transporter for l-aspartate uptake and l-aspartate_ex_/C4DC_in_ antiport, differentiating it from the DcuB-type transporter DcuE for l-malate_ex_/succinate_in_ antiport. Sequence analysis and predicted structural characteristics confirm structural similarity of Asuc_0142 to DcuA, and Asuc_0142 was thus re-named as DcuA_As_. The bovine rumen fluid contains l-aspartate (99.6 µM), whereas fumarate and l-malate are absent. Therefore, bovine rumen colonisers depend on l-aspartate as an exogenous substrate for fumarate respiration. *A. succinogenes* encodes HemG (protoporphyrinogen oxidase) and PyrD (dihydroorotate dehydrogenase) for haem and pyrimidine biosynthesis. The enzymes require fumarate as an electron acceptor, suggesting an essential role for l-aspartate, DcuA_As_, and fumarate respiration for *A. succinogenes* growing in the bovine rumen.

## Introduction

*Enterobacteriaceae* use C4-dicarboxylates (C4DCs) such as fumarate under anaerobic conditions when the oxidative tricarboxylic acid (TCA) cycle is inactive for fumarate respiration [1, 2]. In this pathway, the fumarate is reduced to succinate on the cytoplasmic side of the membrane and succinate is then excreted by fumarate/succinate (or C4DC/succinate) antiport [3, 4]. In most biotopes, fumarate is scarce, and l-malate or l-aspartate are used as precursors [5, 6], which are converted by fumarase or l-aspartase to fumarate. Under anaerobic conditions, *Escherichia coli* and *Salmonella* use the Dcu family of transporters for C4DC/succinate antiport [3, 7, 8]. Dcu consists of the DcuA and DcuB subfamilies which catalyse an exchange of two of C4DCs (C4DC՛_ex_ + C4DC՛՛_in_⇌ C4DC՛_in_ +C4DC՛՛_ex_) where C4DC stands for fumarate, l-malate, l-aspartate or succinate. The members of the Dcu family are 434–446 amino acid residues in length and are predicted to have 11 or 12 transmembrane regions. There are no crystal structures yet available for Dcu transporters. In *E. coli* DcuA is encoded from an operon with the gene for aspartase (*aspA*), and DcuB from an operon with the gene for fumarase B (*fumB*), indicating that their physiological roles are the utilization of l-aspartate and l-malate, respectively. DcuB has a high affinity for l-malate and fumarate and is used for the l-malate/succinate or fumarate/succinate exchange. In *A. succinogenes* a DcuB-type transporter has been termed DcuE [9, 10]. DcuA, on the other hand, has a preference for l-aspartate and catalyses the l-aspartate/succinate exchange [11]. In addition to its role in fumarate respiration, l-aspartate is an important nitrogen source for bacteria. In *Enterobacteriaceae,* l-aspartate is the precursor for several amino acids in addition to the aspartate family of amino acids (Fig. S1A, available in the online version of this article). l-Aspartate provides the ring skeleton in pyrimidine and an amino group for urea and purine synthesis ([12]; an overview in [13]) (Fig. S1B, C). Under some conditions, *E. coli* exploits only the ammonia from l-aspartate as a nitrogen source whereas the carbon backbone is discarded. Thus, under aerobic conditions, when other preferred carbon sources like glycerol are available in combination with l-aspartate, only nitrogen is used for cell growth. The fumarate obtained by deamination of l-aspartate is excreted by an l-aspartate/fumarate exchange that is catalysed by DcuA [11, 13, 14]. The DcuC transporters function in succinate or C4DC export and are distantly related to the DcuA and DcuB transporters [7, 15].

l-Malate and l-aspartate are present in the mouse intestine and represent the actual source for fumarate respiration [5, 6]. Remarkably, fumarate respiration is required for bacterial colonization in the intestine Schubert *et al*., 2021 [5, 6, 16]. The essential role of fumarate respiration is explained by the need for fumarate as an electron acceptor for specific steps of pyrimidine and haem biosynthesis and for redox balancing [13], when O_2_ and nitrate are missing. In enteric bacteria, fumarate respiration is only a minor source of ATP synthesis [13]. This function may be more relevant for other bacteria like *Actinobacillus succinogenes *.

The rumen coloniser *A. succinogenes* produces large amounts of succinate and has been noticed as an industrial succinate producer [17, 18]. The high capacity for succinate production indicates that fumarate respiration plays a major role in the metabolism of *A. succinogenes *. The fumarate for respiration is produced endogenously from hexoses or gained by C4DC uptake from the environment [10]. The bacteria encode several putative carboxylate transporters including the SdcA transporter (Asuc_0304) for the uptake of C4DCs under oxidative conditions [19]. Under anaerobic conditions, six different potential transporters for C4DCs are expressed [10], including two Dcu-type transporters. DcuE (Asuc_1999) is produced constitutively under anaerobic conditions. This transporter is of the DcuB-type and has a strong preference for l-malate and l-malate/C4DC antiport [9, 10]. The second transporter, Asuc_0142, is inducible under anaerobic conditions. It was annotated as a transporter of the DcuB-type as well [18]. The bovine small intestine contains high levels of l-aspartate whereas fumarate and l-malate are hardly detected [20]. This situation may be similar for the bovine rumen fluid where *A. succinogenes* prevails. This situation raises the questions of whether Asuc_0142 is a DcuB-type l-malate/C4DC antiporter (like DcuE) as predicted from annotation and how the need for l-aspartate/C4DC antiport can be satisfied.

In this study, we tested the substrate specificity of Asuc_0142 for C4DCs, and the results revealed a clear preference for l-aspartate in uptake and antiport. In addition, a detailed analysis showed that Asuc_0142 is phylogenetically related more closely to DcuA than to DcuB. Moreover, testing the bovine rumen fluid showed the presence of l-aspartate whereas fumarate and l-malate were essentially lacking. These findings indicate Asuc_0142 has a role in l-aspartate/C4DC antiport in bovine rumen, and Asuc_0142 was renamed as DcuA (or DcuA_As_).

## Methods

### Bacterial strains and growth conditions

The strains and plasmids used in this study are listed in Table 1. Subculture of *Actinobacillus succinogenes* 130Z was grown in Brain Heart Infusion (BHI) medium (Difco, USA) at 37°C for 20–24 h. The main culture was conducted anaerobically in defined medium AM3 [21] with carbon and nitrogen sources: d-glucose (20 mM) (Samchun, South Korea), glycerol (40 mM) (Duksan, South Korea), sodium l-aspartate monohydrate (40 mM) (Sigma-Aldrich, USA), disodium fumarate (40 mM) (Sigma-Aldrich, USA), and NaHCO_3_ (40 mM) (Merck, USA). Strains were anaerobically incubated in rubber-sealed bottles (25 ml medium in 50 ml bottles or 50 ml medium in 250 ml bottles) at 37°C. For gene deletion, *A. succinogenes* 130Z was anaerobically grown in BHI medium. After electroporation, the cell suspension was recovered in BHI with 1 % glucose for 6 h.

**Table 1.** Strains and plasmids used in this study

Strains or plasmids	Relevant genotype	Source/Reference
**Strain**		
* Actinobacillus succinogenes* 130Z	Wild-type strain (DSM 22257)	DSMZ
* Escherichia coli* DH5α	*fhuA2 lac(del)U169 phoA glnV44 Φ80' lacZ(del)M15 gyrA96 recA1 relA1 endA1 thi-1 hsdR17*	Taylor *et al*. (1993) [30]
IMW529	LJ1, *but dcuA::spc^R^, dcuB::kan^R^, dcuC::Tn10(cm^R^), citT::kan^R^, ∆ttdT*	Kim and Unden [22]
LMB070	*A. succinogenes 130Z,* but *ΔAsuc_0142::kan^R^*	This study
**Plasmid**		
pUC19	Cloning vector, amp*^R^*	Enzynomics, Inc.
pKD4	riR, *kan^R^ bla* FRT	Datsenko and Wanner (2000) [31]
pBAD30	*araC, araBAD* promoter, pACYC184, *bla,* amp*^R^*	Guzman *et al*. (1995) [32]
pLS88	Shuttle vector (ATCC 86980), kan*^R^*, str*^R^*, sul*^R^*	ATCC
pMB31	pUC19 with complete gene *sacB* (1422 bp) form pDM4, amp*^R^*	Rhie et al. [10]
pMB61	pBAD30 with Shine-Dalgarno sequence, amp*^R^*	Rhie et al. [10]
pMB147	pMB61 with *Asuc_0142*, amp*^R^*	This study
pMB148	pMB61 with *Ec_dcuA*, amp*^R^*	This study
pMB177	pLS88 with *Asuc_0142*, sul*^R^*, str*^R^*	This study
pMB179	pMB31 with *Asuc_0142*::kan^r^ and *sacB*, amp*^R^*	This study

Subculture of *E. coli* was grown in LB medium (Difco, US) at 37°C for 16 h. For growth test and transport assay, the main culture of *E. coli* was conducted in defined medium M9 [22, 23] at pH 7. Strains were anaerobically grown in rubber-sealed bottles at 37°C. For molecular cloning, *E. coli* was grown in LB aerobically.

### Molecular methods

All plasmids used in cloning were extracted using BIOFACT HiGene Plasmid Mini Prep Kit (BIOFACT, South Korea). Genes were amplified by Phusion High-Fidelity DNA Polymerase (New England Biolabs, USA) in C1000 Thermal Cycler (Bio-Rad, USA).

The deletion of the Asuc_0142 gene in *A. succinogenes* 130Z was performed by homologous recombination using pMB179 as described by Rhie *et al*. [9]. pMB179 was constructed so that the upstream and downstream regions of Asuc_0142 gene were flanking the kanamycin resistance gene derived from pKD4. The PCR product (using Forward primer 5′-CAT ACC CGG GAT GAC TGC AA-3′ and reverse primer 5′-CGC GGA GCT CTT AGA TAA AC-3′) was cloned into the pMB31 plasmid harbouring *sacB* gene by SmaI and SacI restriction enzymes. For homologous recombination, transformation of pMB179 to *A. succinogenes* 130Z was performed by electroporation [9]. The cell suspension was cultured on a BHI plate containing sucrose to remove the plasmid and obtain the Δ0142 strain of *A. succinogenes *. To verify the gene deletion Asuc_0142, gDNA was amplified by PCR using primers del1_for 5′-CGG TTG GCA ATC GCT TGT AA-3′ and del1_rev 5′-ACC TAT ACC ACC GAC CCT TG-3′ (Δ0142, 1.1 kb; wild-type 0142, no PCR products) and primers del1_for and del2_rev 5′-GAG CAG CCG ATT GTC TGT TG-3′ (Δ0142, no PCR product; wild-type 0142, 0.7 kb).

To construct the complementation plasmid (pMB177) for the Δ0142 strain *A. succinogenes* (LMB070), Asuc_0142 was cloned into the pLS88 plasmid containing the replication origin for strains of family *Pasteurellaceae *. The Asuc_0142 gene was amplified with primers pLS88_asuc_0142_EcoRI_For (5′-CTA GCG AAT TCA GGA GGG TGC ACT TAT GAC-3′) and pLS88_asuc_0142_SacI_Rev (5′-CCA AAT GGC GGG AGC TCA ACA AAA AGT G-3′) using genomic DNA of *A. succinogenes* 130Z as template. The PCR product was cloned into the pLS88 plasmid using EcoRI and SacI restriction enzymes.

For cloning the Asuc_0142 gene in the pBAD system (pMB147), the Asuc_0142 gene was amplified with primers asuc_0142_SfoI_For (5′-GGC TGA AAA GGG CGC CTT AC-3′) and asuc_0142_FspI_Rev (5′-CAC TTT TTC CTT TGC GCA GG-3′) using genomic DNA of *A. succinogenes* 130Z as template. The PCR product was cloned into the pMB61 plasmid using SfoI and FspI restriction enzymes (New England Biolabs, USA). For cloning *dcuA* of *E. coli* in the pBAD system (pMB148), the *dcuA* gene was amplified with primers DcuA_SacI_For (5′-CAA GGA GCT CTA ATA TGC TAG TTG TAG AAC TC-3′) and DcuA_SalI_Rev (5′-GAA GGA TGT TAG TCG ACG GGA AAG AAA GC-3′) using genomic DNA of *E. coli* MG1655 as template. The PCR product was cloned into the pMB61 plasmid using SacI and SalI restriction enzymes.

### Transport assays

#### Cell suspension of *A. succinogenes*

Strains 130Z, LMB070, and LMB070 containing pMB177 were anaerobically grown on glucose (20 mM) plus l-aspartate (40 mM) in AM3 medium at 37°C. At OD_600_ 0.4, cells were harvested by centrifugation at 4°C. Harvested cells were washed and suspended in anoxic ice-cold phosphate buffer (100 mM Na_2_HPO_4_/KH_2_PO_4_, 1 mM MgSO_4_, pH 7) to an OD_600_ of 8.0 and subsequently degassed on ice.

#### Cell suspension of *E. coli*

IMW529, IMW529 containing pMB147, and IMW529 containing pMB148 were anaerobically grown on glucose (50 mM) plus l-aspartate (40 mM) in M9 medium l-arabinose (20 µM) at 37°C. At OD_600_ 0.8, cells were harvested by centrifugation at 4°C. Harvested cells were washed and suspended in anoxic ice-cold phosphate buffer (100 mM Na_2_HPO_4_/KH_2_PO_4_, 1 mM MgSO_4_, pH 7) to an OD_600_ of 8.0 and subsequently degassed on ice.

#### General procedures for uptake assay

The cell suspensions of *A. succinogenes,* or of *E. coli,* respectively, were pre-incubated at 37°C, and at this stage lactose (20 mM) was added to *E. coli *. The uptake was started by mixing each of the radio-labelled substrates with cell suspensions of *E. coli* or *A. succinogenes,* respectively, at 37°C. The radio-labelled substrates used are l-[^14^C]aspartate (201 mCi l-[^14^C]aspartic acid mmol^−1^; Moravek Biochemicals, USA), [^14^C]fumarate (9.7 mCi [2,3-^14^C]fumarate mmol^−1^; American Radiolabeled Chemicals Inc., USA), l-[^14^C]malate (55.0 mCi l-[^14^C] malic acid mmol^−1^; American Radiolabeled Chemicals, Inc., USA), [^14^C]succinate (54.0 mCi [1,4-^14^C]succinate mmol^−1^; Moravek Biochemicals, USA). After specific times, the reaction was stopped by adding 0.9 ml ice-cold 0.1 M LiCl to the mixture and quickly vacuum filtering through a membrane filter (mixed cellulose ester, diameter 25 mm, pore size 0.2 µm, A020A025A; ADVANTEC, Japan), and filters were immediately washed twice with the same stop solution. The radioactivity inside the cells was measured by a liquid scintillation counter (Beckman, USA). The uptake amount of substrate was determined by calculating the intracellular radio-labelled substrate, considering that OD_600_ 1.0 of *E. coli* corresponds to 218 mg dry weight/l and *A. succinogenes* 313.8 dry weight/l [10, 24]. The intracellular concentration of substrate in *E. coli* was calculated by the amount of radio-labelled substrate taken up and a cytoplasmic volume of 2.15 ml mg^−1^ dry weight [24].

#### Concentration-dependent assay

First, 50 µl of cell suspension was added to 50 µl of various concentrations of l-[^14^C]aspartate at 37°C. After incubation at 37°C for 1 min, the reaction was stopped and radioactivity was measured as described above.

#### Time-dependent assay

First, 200 µl of cell suspension was added to 200 µl of 200 µM l-[^14^C]aspartate, [^14^C]fumarate, l-[^14^C]malate, [^14^C]succinate at 37°C. After incubation at 37°C for 0, 1, 2, 5 min, 100 µl of the reaction sample was mixed with 0.9 ml stop solution and measured as described above.

#### Competitive inhibition

First, 50 µl of cell suspension was added to 50 µl of 200 µM of l-[^14^C]aspartate or [^14^C]fumarate at 37°C in presence of 1 mM unlabeled competitors (succinate, fumarate, l-malate, l-aspartate, l-asparagine, l-glutamate, α-ketoglutarate, lactose, citrate, butyrate, or oxaloacetate). After incubation at 37°C for 1 min, the reaction was stopped, and the radioactivity was measured as described above.

#### Exchange assay

First, 50 µl of cell suspension was added to 50 µl of 200 µM of l- [^14^C]aspartate, [^14^C]fumarate, or [^14^C]succinate and incubated at 37°C for 10 min. When the uptake was saturated (10 min), 1 mM of unlabeled substrates (succinate, fumarate, l-aspartate, oxaloacetate, or l-tartrate) were added. After 1 and 2 min, the efflux of radio-labelled substrate was determined by the change in intracellular concentration [22].

### Analysis of rumen fluid

Fresh rumen fluid (1 litre) was purchased from Bar Diamond (USA). The analysis of rumen fluid was performed in the National Instrumentation Centre for Environmental Management (NICEM, Agriculture and Life Sciences, Seoul National University, Korea). Free amino acids and organic acids were analysed by HPLC (Dionex Ultimate 3000, Thermo Dionex, USA). Amino acids were separated using VDSpher 100 C18-E column (4.6 mm × 150 mm, VDS Optilab, Germany) and detected by FL (Agilent 1260 Infinity, USA) and UV (338 nm). Mobile phase was composed of A (40 mM sodium phosphate dibasic, pH 7) and B (dH_2_O/acetonitrile/methanol=10 : 45 : 45, v/v %), and the column temperature was 40°C. Organic acids were separated using an Aminex 87 h column (300×10 mm, Bio-Rad) and detected by RI detector (ERC, RefractoMAX520, Japan) and UV (210 nm). The mobile phase was 0.01 H_2_SO_4_ and the column temperature was 40°C. Fatty acids were analysed by Agilent 7890A (Agilent, USA) using column DB-23 (60 mm × 0.25 mm × 0.25 µm, Agilent, USA) and detected with FID (280°C, H_2_ 35, air 350, He 35 ml min^−1^).

## Results and discussion

### l-Aspartate is the preferred substrate for the transport protein Asuc_0142

**Fig. 1.**
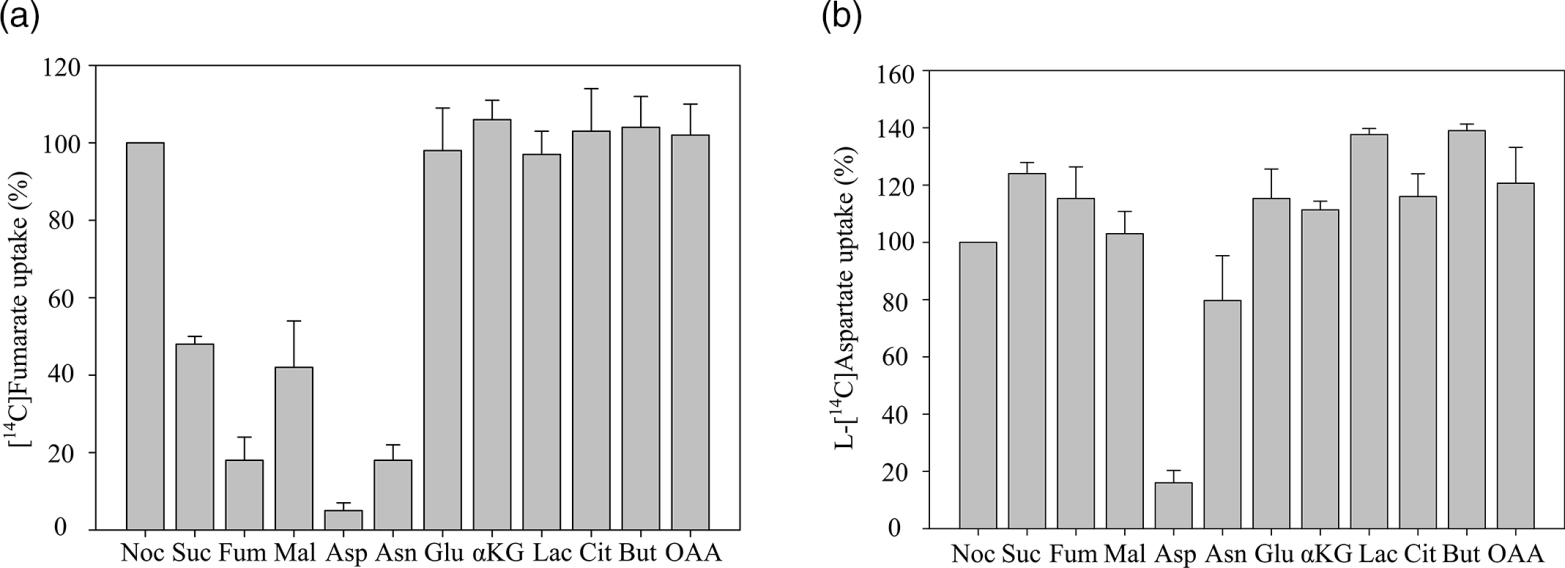
Substrate competition on the uptake of [^14^C]fumarate (**a**) or l-[^14^C]aspartate (**b**) by Asuc_0142. The C4DC-deficient *E. coli* strain IMW529 containing pMB147 (asuc_0142) was grown anaerobically in M9 medium with gluconate (50 mM) and l-aspartate (40 mM) at 37 °C to OD_600_ 0.8. Uptake of 100 µM [^14^C]fumarate (**a**) or l-[^14^C]aspartate (**b**) was determined after 1 min of incubation of anaerobic cell suspension in the presence of unlabeled competitors (1 mM). The uptake activity of 100 % [^14^C]fumarate and 100 % l-[^14^C]aspartate corresponded to 5.52 µmol⸱min^−1^⸱gDW^−1^ and 6.16 µmol⸱min^−1^⸱gDW^−1^, respectively. Competitors: Noc, No competitor; Suc, succinate; Fum, fumarate; Mal, l-malate; Asp, l-aspartate; Asn, l-asparagine; Glu, l-glutamate; αKG, α-ketoglutarate; Lac, lactate; Cit, citrate; But, butyrate; OAA, oxaloacetate. All data are the average of at least three biological replicates using independent growth cultures. Error bars indicate the standard deviation.

Asuc_0142 of *Actinobacillus succinogenes* [18] is annotated as an anaerobic C_4_-dicarboxylate (C4DC) transporter DcuB in DBGET (https://www.genome.jp/dbget/), which is an integrated database system of 18 KEGG and 19 other major biological databases. DcuB is the major fumarate/succinate antiporter of fumarate respiration of *E. coli* [2]. Asuc_0142 produced heterologously in *E. coli* showed significant fumarate uptake (>6 µmol⸱min^−1^⸱gDW^−1^). To avoid interference from endogenous C4DC transporters, *E. coli* IMW529 lacking genes for DcuA, DcuB, DcuC, TtdT, and CitT was used as the test strain for further studies. To identify the substrate specificity of Asuc_0142, the inhibition of [^14^C]fumarate uptake was measured in a substrate competition assay using unlabeled C4DC substrates in addition to [^14^C]fumarate. Dilution of [^14^C]fumarate by a ten-fold excess of unlabeled fumarate decreased the uptake of [^14^C]fumarate to 18 % of the former activity (Fig. 1a). The decrease results from the dilution of the specific radioactivity to approximately 9 % of the former. The expected inhibition of uptake (91 %) is compensated to some extent by the increased overall uptake rate that results from the increased concentration of total fumarate (1100 µM versus 100 µM). An inhibition of 82 % was observed (Fig. 1a), confirming the efficient competition by fumarate. However, a ten-fold excess of l-aspartate yielded an even more potent inhibition by 95 % of [^14^C]fumarate. l-Asparagine caused an inhibition of [^14^C]fumarate uptake comparable to fumarate, whereas the inhibition by succinate and l-malate was only moderate. Other mono-, di-, or tri-carboxylic acids had no effect on [^14^C]fumarate uptake (Fig. 1a).

The substrate preference of Asuc_0142 for the C4DCs was tested in a further experimental design following inhibition of l-[^14^C]aspartate uptake by other C4DCs (Fig. 1b). The l-[^14^C]aspartate uptake was noticeably inhibited (84 %) only by unlabeled l-aspartate, whereas fumarate and any other C4DCs had no significant effect, suggesting that l-aspartate is the preferred substrate for Asuc_0142. l-Asparagine inhibited the l-[^14^C]aspartate uptake with low efficiency (20 %). This effect is considered to be indirect by a periplasmic asparaginase II, which converts l-asparagine to l-aspartate. This point is discussed later in this study.

These findings indicate that the preferred order of substrate uptake by Asuc_0142 is L-aspartate >>fumarate>l-malate and succinate. Remarkably, the inhibition (or competition) by l-malate and succinate is low, suggesting a high preference of l-aspartate.

### Asuc_0142-dependent uptake of l-aspartate in *A. succinogenes*

**Fig. 2.**
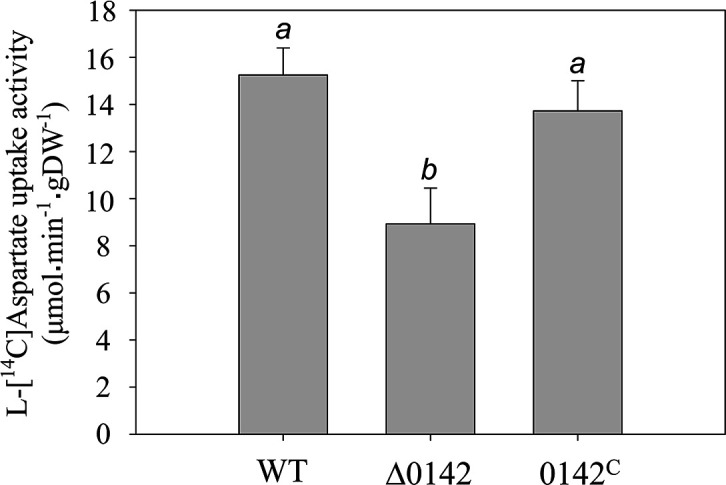
Initial uptake of l-[^14^C]aspartate in *A. succinogenes *. *A. succinogenes* strains [wild-type (WT), LMB070 (Δ0142), and LMB070 complemented with plasmid harbouring Asuc_0142 (0142^C^)] were grown anaerobically in AM3 medium with glucose (20 mM) and l-aspartate (40 mM) (pH7) at 37°C to OD_600_ 0.5. The initial uptake of 200 µM l-[^14^C]aspartate was measured for 20 s. The different superscript indicators reflect significant differences at *P*<0.05. SPSS PAWS Statistics, one-way ANOVA, Duncan’s multiple comparisons. All results are the averages of at least three biological replicates using independent growth cultures. Error bars indicate the standard deviation.

*A. succinogenes* is able to grow under anaerobic conditions in the defined AM3 medium with l-aspartate as a sole nitrogen source (Fig. S2), indicating that l-aspartate can supply the nitrogen for all biosynthetic needs similar to *E. coli* [6]. Growth was not significantly decreased in the Asuc_0142 deletion mutant (Δ0142) (Fig. S2). *A. succinogenes* contains several other potential C4DC transporters such as Asuc_1999 (DcuE), Asuc_1063 (DcuC family), Asuc_0304 (SdcA), Asuc_1568 (DASS family), and Asuc_0270–0273 (TRAP family) in addition to Asuc_0142 [10]. Many C4DC transporters have broad substrate specificity and replace each other functionally when individual transporters are deleted [7, 15]. An effect of Asuc_0142 deletion (Δ0142) was observed; however, when the initial uptake rates were determined after 20 s of transport (Fig. 2). The l-[^14^C]aspartate uptake was significantly decreased by Asuc_0142 deletion. The lowering was complemented after introducing Asuc_0142 (0142^C^) by plasmid expression, confirming that l-aspartate uptake is performed by Asuc_0142.

### Kinetics of l-[^14^C]aspartate uptake by Asuc_0142

**Fig. 3.**
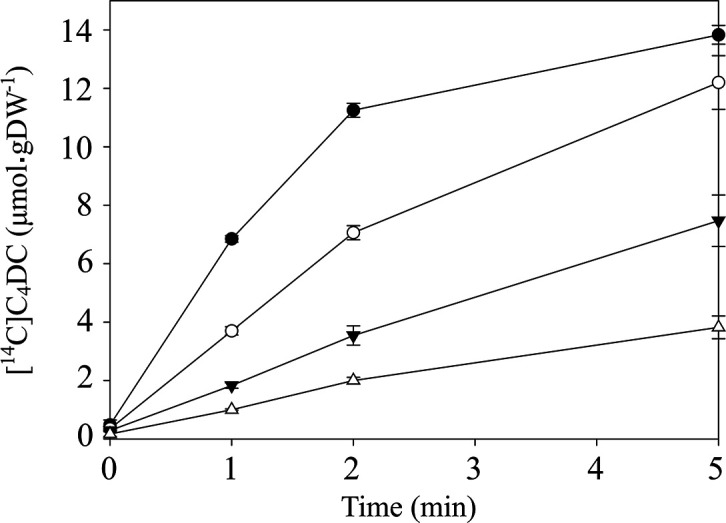
The uptake kinetics of [^14^C]C4DCs by Asuc_0142 in *E. coli* IMW529. Uptake kinetics of 100 µM of l-[^14^C]aspartate (●), [^14^C]fumarate (○), l-[^14^C]malate (▼), and [^14^C]succinate (△) was analysed in cell suspensions of the C4DC-deficient *E. coli* strain IMW529 containing pMB147 (asuc_0142). Growth was performed in M9 with gluconate (50 mM) and l-aspartate (40 mM) under anaerobic conditions to OD_600_ 0.8. All results are the averages of at least three biological replicates using independent growth cultures. Error bars indicate the standard deviation.

To avoid interference from the other potential C4DC transporters of *A. succinogenes *, and for more clear-cut data, further experiments on Asuc_0142 were conducted in the *E. coli* strain (IMW529) lacking the anaerobic C4DC transporters DcuA, DcuB, DcuC, TtdT and CitT [22]. The uptake kinetics of Asuc_0142 was analysed in *E. coli* IMW529 producing Asuc_0142. With 100 µM of l-[^14^C]aspartate, the initial uptake was high (6.85 µmol⸱gDW^−1^ after 1 min), and uptake was almost saturated after 2 min (Fig. 3). Under the same conditions, the uptake of other C4DCs was lower, in the order of fumarate, l-malate, and succinate, showing that Asuc_0142 has the highest transport activity for l-aspartate.

**Fig. 4.**
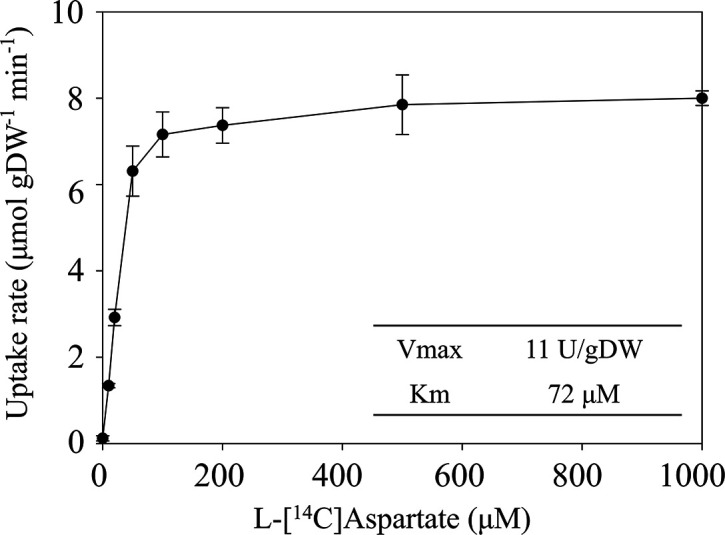
The concentration-dependent uptake of l-[^14^C]aspartate by Asuc_0142 in *E. coli* IMW529. The C4DC-deficient *E. coli* strain IMW529 containing pMB147 (asuc_0142) was grown anaerobically in M9 with gluconate (50 mM) and l-aspartate (40 mM) at 37 °C to OD_600_ 0.8. The 1 min uptake of l-[^14^C]aspartate was determined with concentrations of 0, 10, 20, 50, 100, 200, 500, and 1000 µM. All results are the averages of at least three biological replicates using independent growth cultures. Error bars indicate the standard deviation.

The concentration dependence of l-aspartate uptake by Asuc_0142 was analysed in the *E. coli* strain similar to a Michaelis-Menten-kinetics. The uptake after 1 min was measured, and the maximal uptake rate was already reached at a low concentration (100 µM) of l-[^14^C]aspartate (Fig. 4). The maximum uptake rate (V_max_) was 11.7 µmol⸱min^−1^⸱gDW^−1^ and the K_m_ value 72.6 µM l-aspartate, confirming that Asuc_0142 has a high affinity for l-aspartate.

### Bidirectional l-aspartate/C4DC exchange by Asuc_0142

**Fig. 5.**
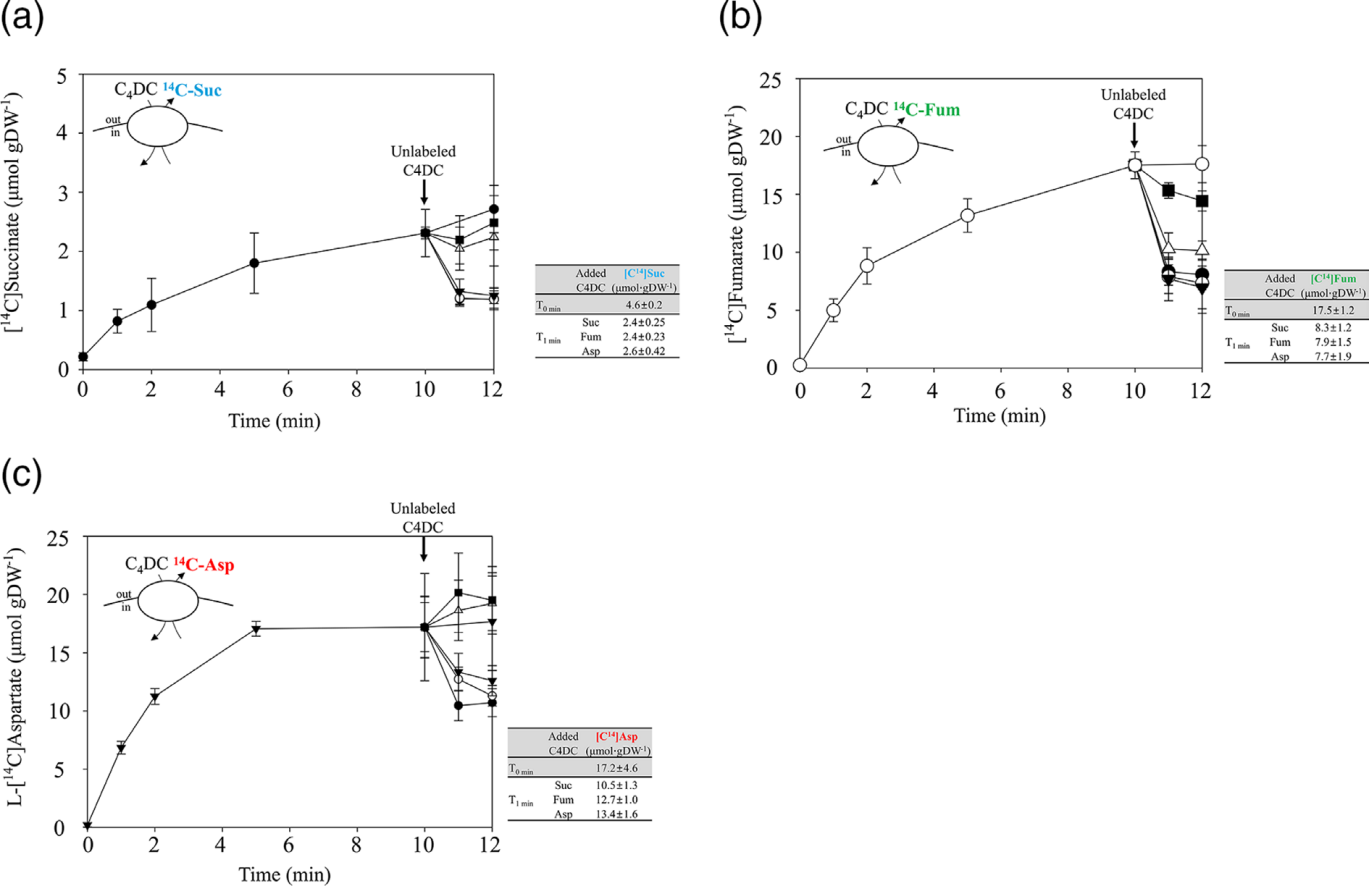
Efflux of [^14^C]succinate (**a**), [^14^C]fumarate (**b**), and l-[^14^C]aspartate (**c**) from *E. coli* cells by Asuc_0142 upon addition of external C4DC. Degassed cell suspension of *E. coli* IMW529 containing pMB147 (asuc_0142) were incubated with 100 µM [^14^C]succinate (**a**), 100 µM [^14^C]fumarate (**b**), or 100 µM l-[^14^C]aspartate (**c**). After 10 min uptake of each [^14^C]-labelled substrate, 1 mM of unlabeled succinate (●), fumarate (○), aspartate (▼), oxaloacetate (△), or tartrate (■) was added (↓). The decrease of the intracellular [^14^C]substrate due to the addition of external substances was determined by filtration assay. The value of *E. coli* IMW529 without plasmid was used as the blank value. All results are the averages of at least three biological replicates using independent growth cultures. Error bars indicate the standard deviation.

The Asuc_0141 gene that is upstream of the gene for the l-aspartate transporter Asuc_0142 is predicted to encode an l-aspartate ammonia-lyase that deaminates l-aspartate to fumarate (https://www.genome.jp/dbget-bin/www_bget?asu:Asuc_0141). Ammonia may serve as a nitrogen source and fumarate as an electron acceptor in fumarate respiration. Transcriptome analysis performed by Rhie *et al*. [10] revealed the induction of Asuc_0142 under anaerobic growth on glucose. Therefore, Asuc_0142 is considered to function as an l-aspartate_ex_/succinate_in_ antiporter for fumarate respiration. To verify this type of antiport, the *E. coli* cells with Asuc_0142 were loaded with [^14^C]succinate. Then unlabeled l-aspartate (or other C4DCs) were added, and succinate efflux accompanying the l-aspartate addition was measured. When 100 µM [^14^C]succinate were used for loading, the cells contained 4.6 µmol⸱gDW^−1^ after saturation, corresponding to 2.14 mM intracellular [^14^C]succinate. Approximately half of the accumulated [^14^C]succinate was released rapidly from the cells after the addition of l-aspartate, succinate, or fumarate. The efflux rate of [^14^C]succinate was approximately 2 µmol⸱min^−1^⸱gDW^−1^ (Fig. 5a, Table S1A). If measured under more defined conditions, it can be estimated that the efflux rate has reached a level similar to the l-aspartate uptake rate of about 8 µmol⸱min^−1^ gDW^−1^ determined at a substrate concentration of 1 mM (Fig. 4). This estimation indicates that the l-aspartate_ex_/C4DC_in_ antiport occurs at high rates and is comparable to the uptake rate.

Strecker *et al*. [11] showed that DcuA of *E. coli* functions as an l-aspartate/fumarate antiporter, when the major role of l-aspartate is the supply of nitrogen (rather than supply of fumarate). The l-aspartate/fumarate antiport results in the net uptake of ammonia as a nitrogen source. To test this type of transport, cells loaded with [^14^C]fumarate (17.5 µmol⸱gDW^−1^ under saturating conditions, corresponding to 8.14 mM intracellular [^14^C]fumarate) performed efflux of [^14^C]fumarate after addition of succinate, fumarate, and l-aspartate with high rates (approximately 9~10 µmol⸱min^−1^⸱gDW^−1^) (Fig. 5b, Table S1B). The high rates of [^14^C]fumarate efflux shown in Fig. 5b exceed those for [^14^C]succinate efflux shown in Fig. 5a. The difference may be explained in part by the higher levels of intracellular [^14^C]fumarate (8.14 mM) compared with those of [^14^C]succinate (2.14 mM). From the succinate and fumarate, approximately 50 % was released from the cell in the first 1 min (Fig. 5b, Table S1).

The test in the reverse direction revealed that Asuc_0142 also catalyses the efflux of intracellular l-[^14^C]aspartate when succinate, fumarate, or l-aspartate are provided from the outside. The efflux rate was approximately half of the [^14^C]fumarate efflux (Fig. 5c, Table S1C).

Together, these results indicate that Asuc_0142 transports C4DCs l-aspartate, fumarate and succinate from the outside to inside (uptake mode) and from the inside to the outside in a bidirectional C4DC/C4DC exchange. Here, l-aspartate serves as the best substrate for uptake, and succinate and fumarate are the preferred internal substrates for l-aspartate/C4DC efflux.

### Asuc_0142 is a DcuA-type transporter

Asuc_0142 and DcuE of *A. succinogenes* show 84 and 44% sequence identity to DcuB of *Haemophilus influenzae *, respectively, which was used to arrange Asuc_0142 in the DcuB-type [18]. However, the sequence identity of Asuc_0142 to DcuA and DcuB of *E. coli* is very similar (43 and 41% identity, respectively) (Fig. S3), and sequence comparison between Asuc_0142 and a large number of transporters of the Dcu family confirmed a closer relation of Asuc_0142 to the DcuA than the DcuB transporters (Fig. S4). Accessibility and topology studies revealed 12 transmembrane (TM) helices for DcuB of *E. coli* [25]. DeepTMHMM, an advanced protein topology prediction database based on a deep learning algorithm (https://dtu.biolib.com/DeepTMHMM), confirmed the presence of 12 TM helices for DcuB. The same number of TM helices (12) were predicted by DeepTMHMM for DcuE in accordance with the DcuB-type function [9]. DeepTMHMM suggested 13 TM helices for DcuA, for which genetic topology studies are available [26], and the same number was predicted for Asuc_0142, suggesting a different TM topology for DcuA and DcuB and a similar topology for Asuc_0142 and DcuA.

**Fig. 6.**
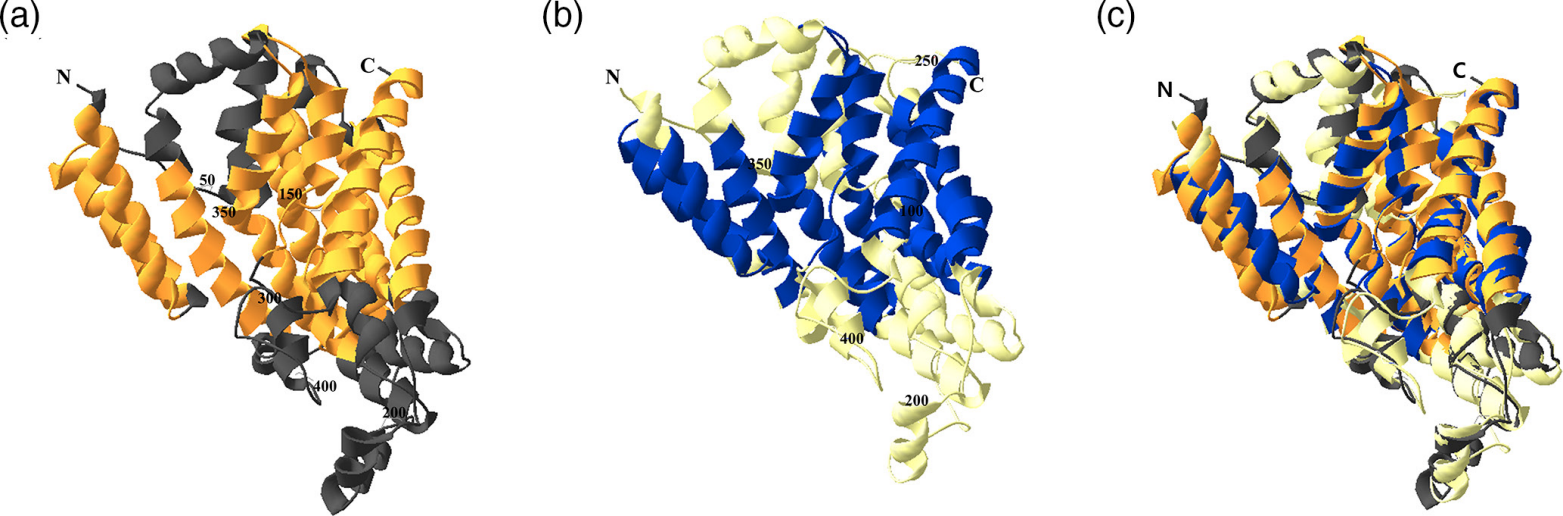
Comparison of predicted tertiary structures of Asuc_0142 and DcuA of *E. coli *. The protein structures of Asuc_0142 (**a**) and DcuA of *E. coli* (**b**) were predicted using RoseTTAFold at Robetta (https://robetta.bakerlab.org/). Pairwise structure alignments (**c**) were performed at RCSB PDB (https://www.rcsb.org/) using jFATCAT (rigid) parameter. TM-score (template modelling score) was 0.91 and Root Mean Square Deviation (RMSD) was 2.00 Å. The SWISS PDB Viewer [33] was used to visualize results. Orange/blue, transmembrane regions; grey/yellow, periplasmic or cytoplasmic regions; N, N-terminus; C, C-terminus.

**Fig. 7.**
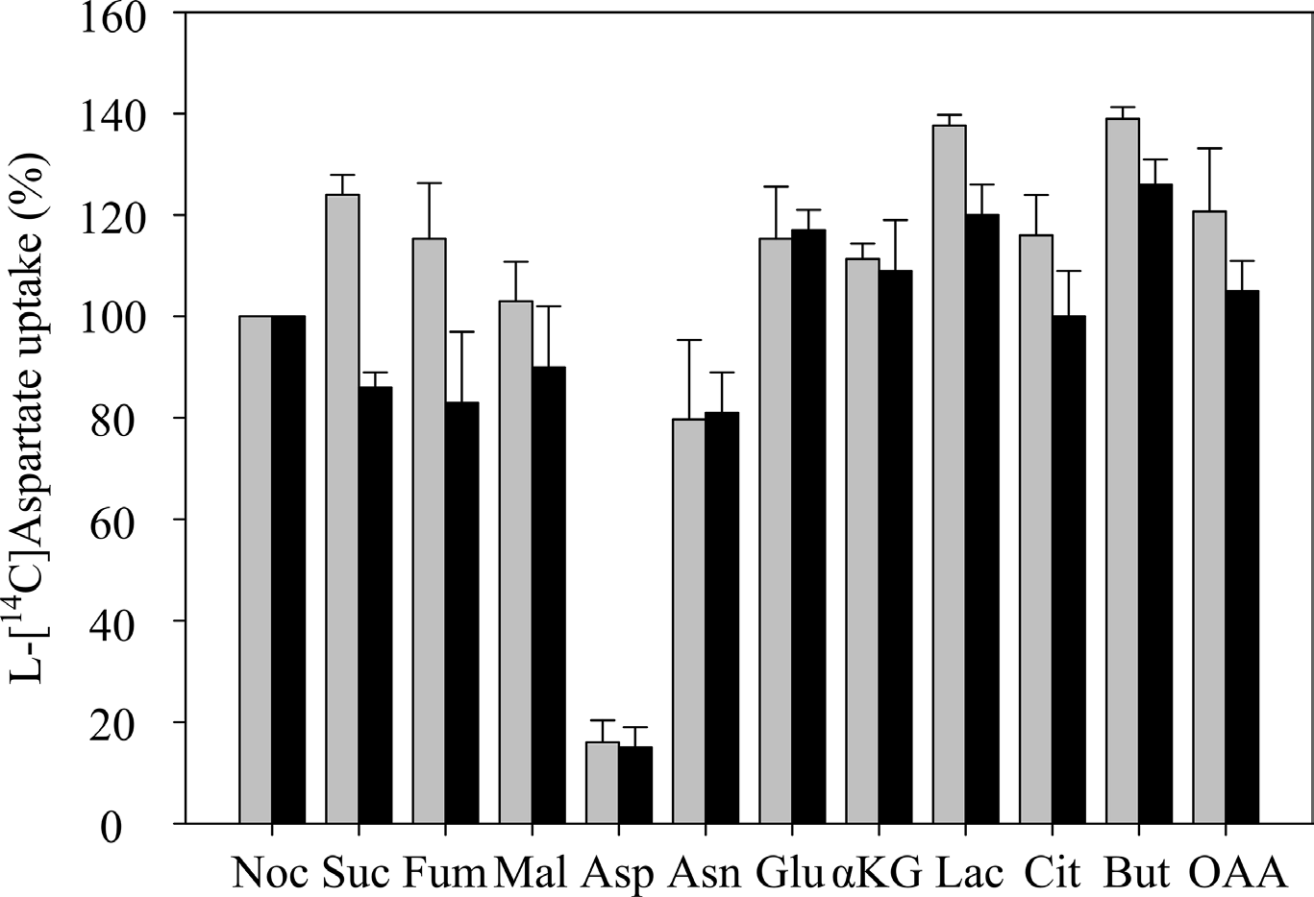
Comparison of substrate competition on the uptake of l-[^14^C]aspartate by Asuc_0142 (grey) and DcuA (black). The C4DC-deficient *E. coli* strain IMW529 with the DcuA expression plasmid pMB148 was grown anaerobically. Uptake was tested as described for Fig. 2 for the uptake of 100 µM l-[^14^C]aspartate without and in the presence of competitors (1 mM) and compared with the values of Asuc_0142 of Fig. 2b. The 100 % uptake rate of DcuA was 8.57 µmol gDW^−1^ min^−1^ and that of Asuc_0142 was 6.16 µmol gDW^−1^ min^−1^. Competitors: Noc, No competitor; Suc, succinate; Fum, fumarate; Mal, l-malate; Asp, l-aspartate; Asn, l-asparagine; Glu, l-glutamate; αKG, α-ketoglutarate; Lac, lactate; Cit, citrate; But, butyrate; OAA, oxaloacetate. All results are the averages of at least three biological replicates using independent growth cultures. Error bars indicate the standard deviation.

The predicted overall tertiary structures of Asuc_0142 and DcuA of *E. coli* were also compared (Fig. 6). There are no experimental structures of transport proteins for the Dcu family. Therefore, the structures of Asuc_0142 and DcuA were predicted using RoseTTAFold at Robetta (https://robetta.bakerlab.org/) (Fig. 7a, b). The structures were then analysed by pairwise structure alignment on RCSB PDB (https://www.rcsb.org/) using jFATCAT (rigid) parameter (Fig. 7c). The resulting TM-score (template modelling score) was 0.91, and the Root Mean Square Deviation (RMSD) 2.00 Å, indicating that both proteins have the same fold (TM-score >0.5) [27] and are homologous (RMSD <3 Å) [28].

Together these findings suggest that Asuc_0142 can be classified based on sequence evaluation with predicted TM topology and predicted overall structure as a DcuA-type transporter, which is in very good agreement with its biochemical properties, with the preference for l-aspartate and l-aspartate_ex_/C4DC_in_ antiport. The latter differentiates DcuA from DcuB, which has a preference for l-malate (or fumarate) and l-malate_ex_/C4DC_in_ antiport [4, 11] (Fig. 7). Additionally, the K_m_ values of Asuc_0142 (72 µM, see Fig. 4) and DcuA (43 µM [11]) for l-aspartate are similar. Therefore, Asuc_0142 was renamed as DcuA_As_ (DcuA in *A. succinogenes*).

The bovine rumen fluid contains l-aspartate (99.6 µM), whereas fumarate and l-malate are absent. Therefore, bovine rumen colonisers depend on l-aspartate as an exogenous substrate for fumarate respiration. *A. succinogenes* encodes HemG (protoporphyrinogen oxidase) and PyrD (dihydroorotate dehydrogenase) for haem and pyrimidine biosynthesis, which require fumarate respiration, suggesting an essential role for l-aspartate, DcuA_As_, and fumarate respiration for *A. succinogenes* growing in the bovine rumen.

### DcuA_As_ provides *A. succinogenes* with l-aspartate abundant in the bovine rumen as an electron acceptor for fumarate respiration

**Fig. 8.**
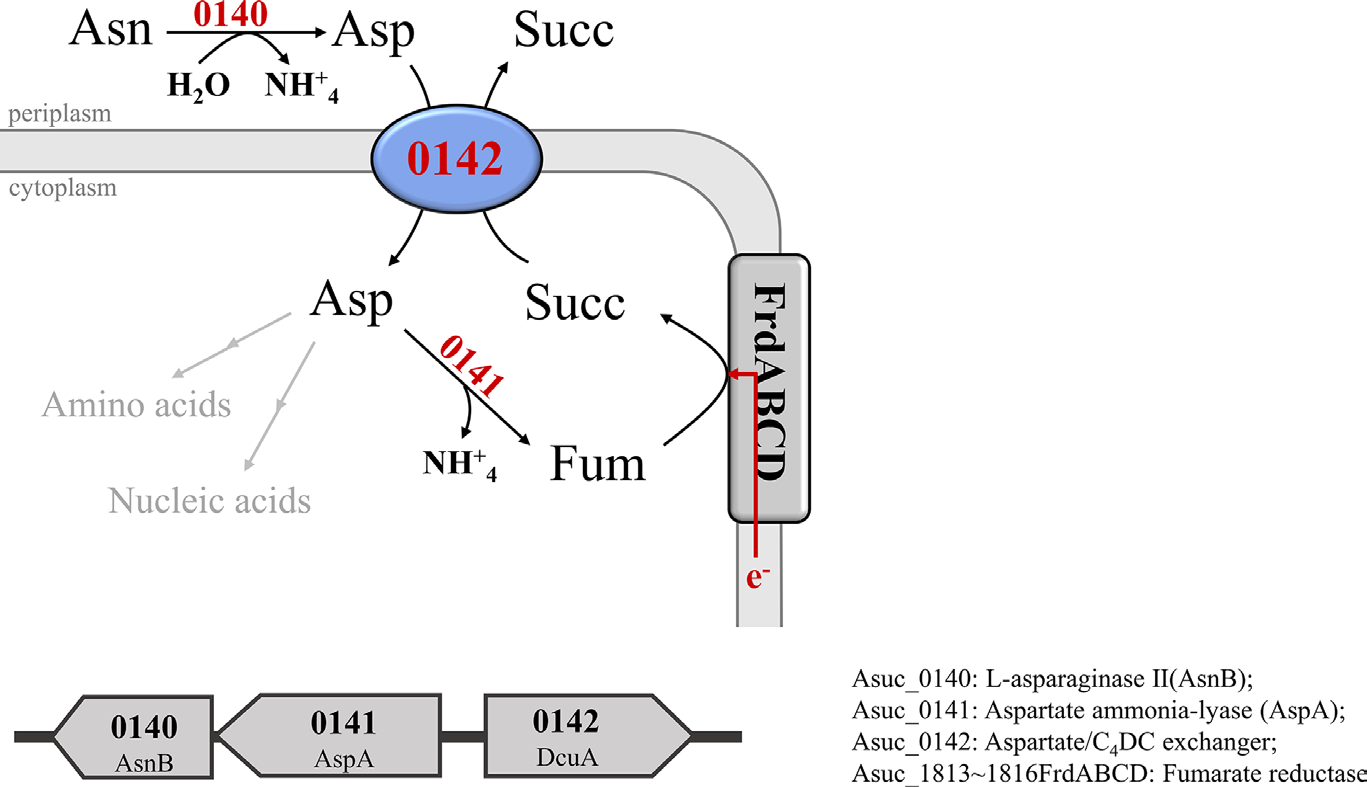
Scheme for l-asparagine and l-aspartate metabolism by *A. succinogenes:* roles as a nitrogen source, as a precursor of fumarate, and in pH homeostasis. The gene cluster Asuc_0140, Asuc_0141 and Asuc_0142 encodes a putative periplasmic asparaginase II (AsnB), a putative l-aspartase (AspA), and DcuA. The reactions and the cellular locations of the enzymes are shown. l-Aspartate is used as a precursor, building block, or source of ammonia for other nitrogen-containing components (compare Fig. 1, or [13]). Fumarate is used as an electron acceptor for fumarate respiration.

Fumarate respiration appears to be important for energy conservation by *A. succinogenes* that inhabits the anaerobic rumen. Fumarate and l-malate are not detectable in bovine rumen fluid (fumarate <0.23 mg l^−1^, l-malate 0.27 mg l^−1^), while high and significant levels of l-aspartate (13.26 mg l^−1^) and l-asparagine (1.08 mg l^−1^) were found (Table 2) . Thus, l-aspartate is the amino acid with the second highest level in the rumen fluid after l-glutamate. The genes Asuc_0140 and Asuc_0141 with homology to a periplasmic asparaginase II (AnsB) and aspartate ammonia-lyase (AspA) are located upstream of Asuc_0142 (Fig. 8). According to the scheme in Fig. 8, periplasmic asparaginase II (Asuc_0140) deamidates l-asparagine to l-aspartate, which then enters cytoplasm by the l-aspartate/C4DC exchanger DcuA_As_ (Asuc_0142). Cytoplasmic l-aspartate is subsequently deaminated to fumarate by aspartase (Asuc_0141), and the fumarate serves as an electron acceptor in fumarate respiration. Therefore, l-aspartate (and l-asparagine) are the actual substrates for fumarate respiration in the bovine rumen. l-Aspartate (and l-asparagine) from the rumen are the substrates for fumarate respiration by *A. succinogenes *, in addition to fumarate produced endogenously from hexoses catabolism [19]. This situation is similar to that in the mouse intestine, where l-aspartate and l-malate are the actual substrates for exogenous fumarate respiration Schubert *et al*., 2021 [5, 6].

**Table 2.** The contents of free amino acids (A), organic acids (B), and fatty acids (C) in the bovine rumen fluid (Sterilized Rumen Fluid, #SRF, BarDiamond, Inc. Parma, Indaho USA)

(A)				
**Amino acid**	**Amount**	**Relative amount**	**Molarity**	**Mole ratio**
	**(mg l^−1^)**	**(%)**	**(μM)**	**(%)**
l-Glutamic acid	26.30	32.37	178.75	28.46
l-Aspartic acid	13.26	16.32	99.63	15.86
l-Alanine	9.02	11.10	101.28	16.13
l-Lysine	5.75	7.08	39.33	6.26
l-Arginine	4.65	5.72	26.71	4.25
l-Leucine	3.01	3.70	22.93	3.65
l-Valine	2.84	3.49	24.21	3.86
l-Serine	2.57	3.16	24.42	3.89
l-Threonine	2.40	2.95	20.14	3.21
l-Tyrosine	1.86	2.29	10.29	1.64
Glycine	1.81	2.23	24.12	3.84
l-Isoleucine	1.68	2.07	12.80	2.04
l-Phenylalanine	1.55	1.90	9.36	1.49
l-Proline	1.46	1.79	12.64	2.01
l-Asparagine	1.08	1.33	8.19	1.30
l-Methionine	1.03	1.27	6.90	1.10
l-Histidine	1.00	1.23	6.43	1.02
l-Cystein	nd	nd	nd	nd
l-Glutamine	nd	nd	nd	nd
l-Tryptophan	nd	nd	nd	nd
Total	81.26	100.00	628.14	100.00
(B)				
**Organic acid**	**Amount**	**Molarity**	**Mole ratio**	
	**(mg l^−1^)**	**(mM)**	**(%)**	
Acetic acid	3884.14	88.18	72.81	
Propionic acid	1293.03	17.45	14.41	
Isobutyric acid	396.11	4.50	3.71	
Butyric acid	789.64	8.96	7.40	
Valeric acid	207.35	2.03	1.68	
Fumaric acid	nd	nd	nd	
l-Malic acid	nd	nd	nd	
Succinic acid	nd	nd	nd	
Total	6570.27	121.12	100.02	
(C)				
**Fatty acid**	**Amount**	**Molarity**	**Mole ratio**	
	**(mg g^−1^)**	(mM)*	**(%)**	
Lauric acid	0.22	1.12	0.79	
Palmitic acid	10.10	39.39	27.78	
Stearic acid	24.48	86.05	60.69	
Linoleic acid	2.02	7.20	5.08	
α-Linolenic acid	1.62	5.82	4.10	
Arachidic acid	0.43	1.38	0.97	
Lignoceric acid	0.31	0.84	0.59	
Total	39.18	141.80	100.00	

nd, not detectable

(A)L-cysteine < 0.57 mg l^−1^, l-glutamine < 0.59 mg l^−1^, l-tryptophan < 1.59 mg l^−1^

(B)fumarate < 0.232 mg l^−1^, l-malic acid < 0.268 mg l^−1^, succinic acid < 0.236 mg l^−1^

*Molarity was calculated on the premise that the density of rumen fluid was 1 g ml^−1^.

Fermentation of hay and polysaccharides in the rumen is prone to acidification, which is fatal to cattle. The growth of *A. succinogenes* is also inhibited below pH 6 (data not shown). The release of ammonia when l-aspartate and l-asparagine are used for fumarate respiration could be important for pH neutralization during sugar fermentation.

The concentrations of l-aspartate and l-asparagine (108 µM) are in the same range in the rumen as in the mouse colonSchubert *et al*., 2021 [5, 6]. Fumarate respiration based on l-aspartate and l-malate in the mouse intestine is essential for the colonization of the intestine by *Enterobacteriaceae* [16] and Schubert *et al*., 2021 [5, 6, 29]. *Enterobacteriaceae* require fumarate, first of all, as an electron acceptor in the biosynthesis of haem (HemG protein) and pyrimidine (PyrD protein) and disulphide formation in periplasmic proteins (DsbAB proteins), whereas the ATP gain by fumarate respiration is of minor significance [13]. The *A. succinogenes* genome also encodes HemG (protoporphyrinogen oxidase Asuc_0513) and PyrD (dihydroorotate dehydrogenase Asuc_1385) proteins that are required in *E. coli* under anaerobic conditions using fumarate as an electron acceptor for their function. Overall, the situation for *A. succinogenes* may resemble those described for *E. coli* and *Salmonella,* in which fumarate reduction is essential in haem and pyrimidine synthesis [13] or the enterohaemorrhagic *Escherichia coli* O157:H7 (EHEC), which uses l-aspartate for *de novo* pyrimidine synthesis [20], and l-aspartate has been suggested to provide an ecological niche for the bacteria in the bovine small intestine.

## Conclusion

l-Aspartate (and l-asparagine) in bovine rumen present an abundant source for fumarate respiration by *A. succinogenes *. Uptake is mediated by the l-aspartate/C4DC antiporter DcuA_As_ (Asuc_0142), and the intracellular l-aspartate can be converted to fumarate by aspartate ammonia-lyase (Asuc_0141). The l-asparagine may be transformed by periplasmic asparaginase II (Asuc_0140) to l-aspartate prior to transport. Fumarate serves in addition to its role in fumarate respiration as an essential electron acceptor in haem and pyrimidine biosynthesis. Therefore, DcuA_As_ and l-asparate play an essential role for growth of A. succinogenes in bovine rumen.



## Supplementary Data

Supplementary material 1
