# Supplementary material for: Asuc_0142 of Actinobacillus succinogenes 130Z is the l-aspartate/C4-dicarboxylate exchanger DcuA
Source: Microbiology (Reading). 2023 Oct 31;169(10):001411. doi: 10.1099/mic.0.001411 (PMC10634366; doi:10.1099/mic.0.001411)
Supplement: Supplementary material 1 [file mic-169-1411-s001.pdf]

Fig. S1

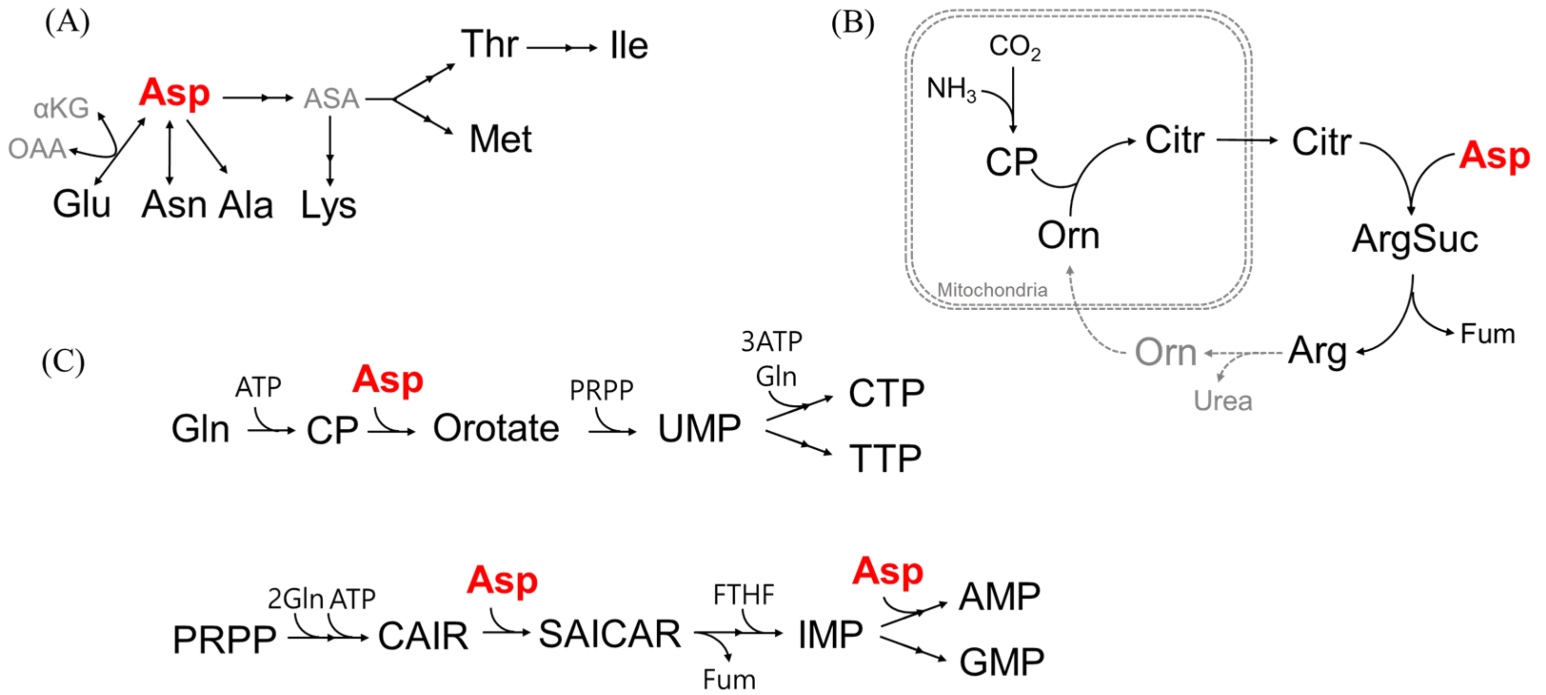

Figure S1. Important metabolism steps requiring aspartate. Aspartate is used (A) as precursor for seven other amino acids, (B) to provide the second amino group for urea formation in the Krebs-Henseleit cycle, and (C) in nucleic acid formation. αKG, α-ketoglutarate; AMP, adenosine monophosphate; ArgSuc, argininosuccinate; ASA, aspartate semialdehyde; CAIR, aminoimidazole carboxylate ribosyl-5-phosphate; Cit, citrulline; CP, carbamoyl phosphate; FTHF, N^{10}-formyl-H_4 folate; Fum, fumarate; GMP, guanidine monophosphate; IMP, inosine monophosphate; OAA, oxaloacetate; Orn, ornithine; PRPP: phosphoribosyl pyrophosphate; SAICAR, aminoimidazole succinyl carboxamide ribosyl-5-phosphate; UMP, uridine monophosphate

Fig. S2

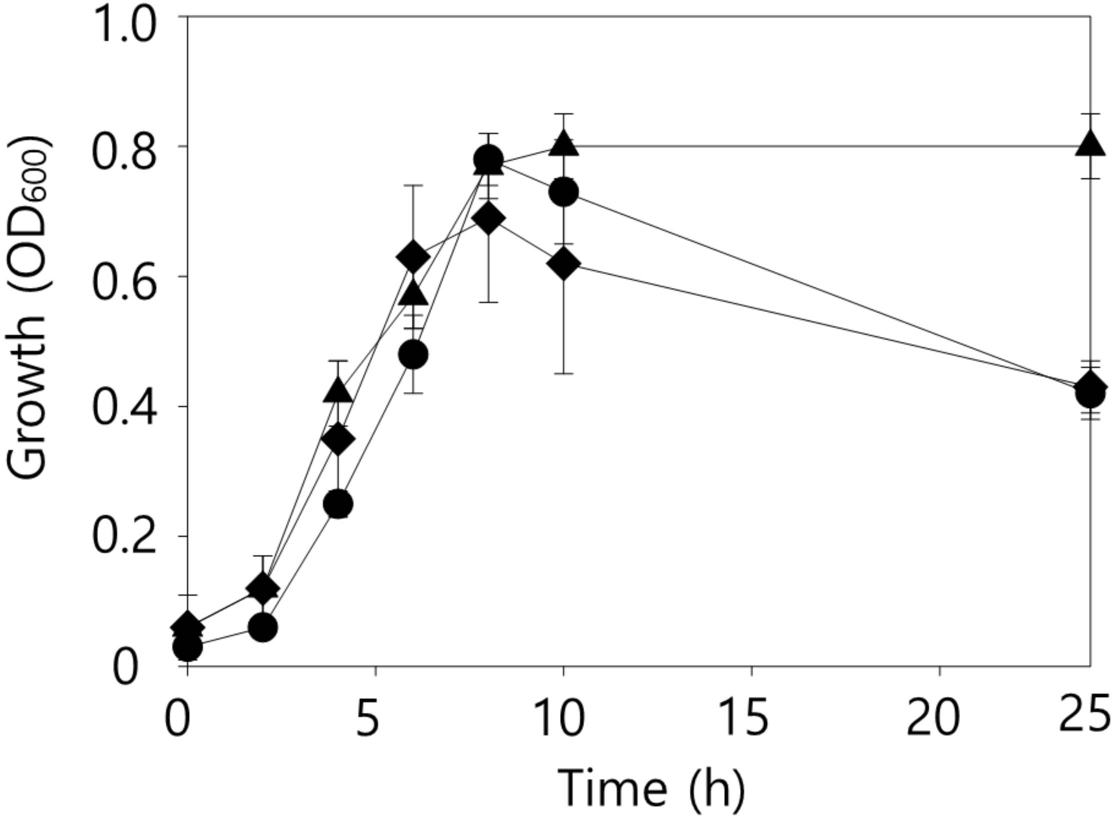

Figure S2. Anaerobic growth of *A. succinogenes.* *A. succinogenes* 130Z strains of wild-type (▲), LMB070 (△0142), or LMB070 with pMB177 (pLS88::asuc_0142) (●) were grown anaerobically in AM3 medium (pH 7) with glucose (20 mM) and aspartate (40 mM) at 37℃. All results are the averages of at least three independent assay series. Error bars indicate the standard deviation.

Fig. S3

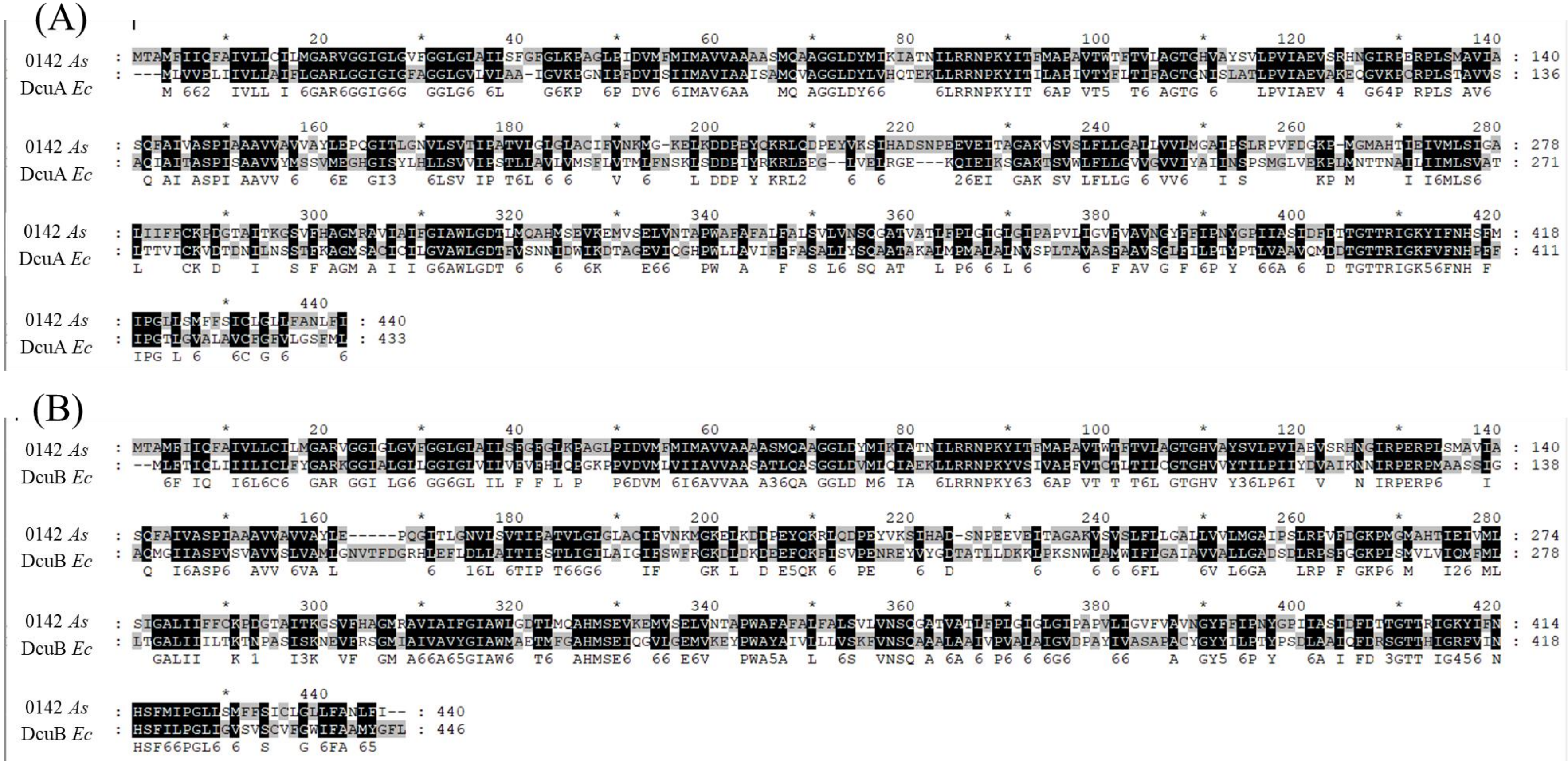

Figure S3. The primary sequence analysis of Asuc_0142. The amino acid sequence of Asuc_0142 was compared to that of (A) DcuA and (B) DcuB of *E. coli* using GeneDoc (Nicholas *et al*., 1997)

Fig. S4

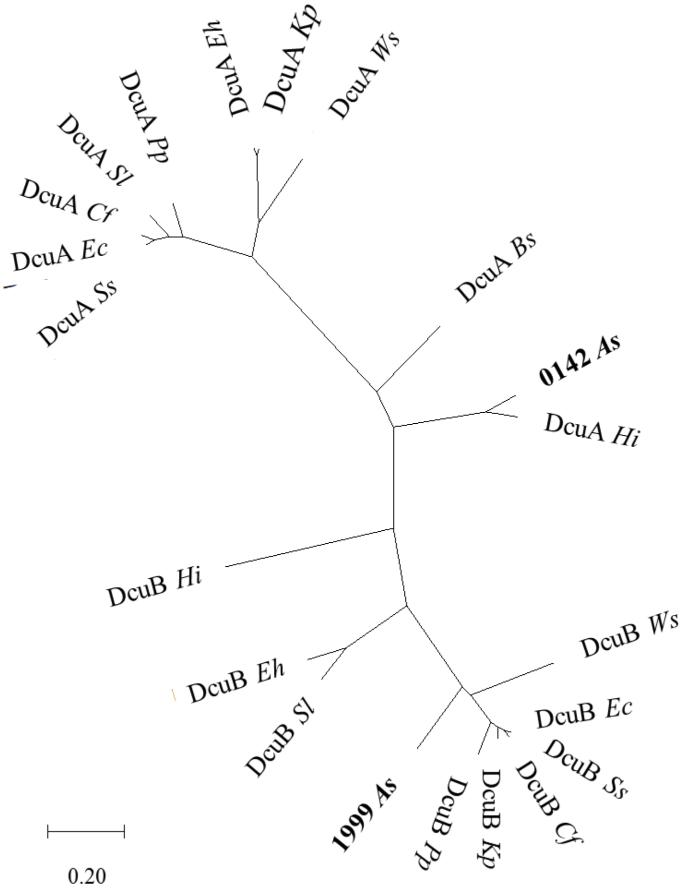

Figure S4. Phylogenic relationship of DcuA-type and DcuB-type transporters in the Dcu family. The phylogenetic tree was inferred by using the Maximum Likelihood method and JTT matrix–based model (Jones *et al.*, 1992). The tree with the highest log likelihood (-9580.69) is shown. Initial tree(s) for the heuristic search were obtained automatically by applying Neighbor-Join and BioNJ algorithms to a matrix of pairwise distances estimated using the JTT model and then selecting the topology with superior log likelihood value. The tree is drawn to scale, with branch lengths measured in the number of substitutions per site. This analysis involved 21 amino acid sequences. There were a total of 561 positions in the final dataset. Evolutionary analyses were conducted in MEGA11 (Tamura *et al.*, 2021).

As, Actinobacillus succinogenes; Bs, Basillus subtilis; Cf, Citrobacter freundii; Ec, Escherichia coli; Eh, Enterobacter hormaechei; Hi, Haemophilus influenzae; Kp, Klebsiella pneumoniae; Pp, Proteus penneri; Sl, Serratia liquefaciens; Ss, Shigella sonnei; Ws, Wolinella succinogenes

Table S1. The intracellular [^{14}C]succinate (A), [^{14}C]fumarate (B), and [^{14}C]aspartate (C) after efflux by Asuc_0142. Cell suspension of the *E. coli* IMW529 containing Asuc_0142 (in plasmid pMB147) was saturated with 100 µM of [^{14}C]succinate, [^{14}C]fumarate, or [^{14}C]aspartate for 10 min. The decrease of radioactivity by addition of unlabeled C_4DCs was measured for 1 min or 2 min. All results are the averages of at least three independent assay series. Suc, succinate; Fum, fumarate; Asp, L-aspartate; OAA, oxaloacetate; Tart, L-tartrate. ND, not determined. Efflux amount (mmol gDW^{-1}) for 1 or 2 min. The data in parentheses indicate the percentage amount of [^{14}C]C4DC excreted from the cell. ND, not detected

(A)

Intracellular accumulated [^{14}C]Suc: 4.6 µmol·gDW^{-1}		Intracellular [^{14}C]Suc (µmol·gDW^{-1})	
		1 min	2 min
	None	ND	5.4±0.8
Added unlabeled C_4DC	Suc	2.4±0.2 (48%)	2.4±0.4 (48%)
	Fum	2.4±0.2 (48%)	2.4±0.3 (48%)
	Asp	2.6±0.4 (43%)	2.5±0.3 (46%)
	OAA	4.1±0.7 (11%)	4.5±0.9 (0%)
	Tart	4.4±0.8 (4%)	5.0±0.9 (0%)

(B)

Intracellular accumulated [^{14}C]Fum: 17.5 µmol·gDW^{-1}		Intracellular [^{14}C]Fum (µmol·gDW^{-1})	
		1 min	2 min
	None	ND	17.6±1.6
Added unlabeled C_4DC	Suc	8.3±1.2 (53%)	8.1±1.3 (54%)
	Fum	7.9±1.5 (55%)	7.3±2.6 (58%)
	Asp	7.7±1.4 (56%)	7.0±1.8 (61%)
	OAA	10.3±1.4 (41%)	10.1±0.8 (42%)
	Tart	15.3±0.7 (12%)	14.4±0.8 (18%)

(C)

Intracellular accumulated L-[^{14}C]Asp: 17.2 µmol·gDW^{-1}		Intracellular L-[^{14}C]Asp (µmol·gDW^{-1})	
		1 min	2 min
	None	ND	17.7±4.2
Added unlabeled C_4DC	Suc	10.5±1.3 (39%)	10.7±1.2 (38%)
	Fum	12.7±1.0 (25%)	11.3±0.8 (34%)
	Asp	13.5±1.6 (22%)	12.6±1.3 (27%)
	OAA	18.6±2.6 (0%)	19.2±2.3 (0%)
	Tart	20.2±2.4 (0%)	19.5±2.9 (18%)
